# Antibody accessibility determines location of spike surface mutations in SARS-CoV-2 variants

**DOI:** 10.1371/journal.pcbi.1010822

**Published:** 2023-01-24

**Authors:** Sören von Bülow, Mateusz Sikora, Florian E. C. Blanc, Roberto Covino, Gerhard Hummer

**Affiliations:** 1 Department of Theoretical Biophysics, Max Planck Institute of Biophysics, Frankfurt am Main, Germany; 2 Malopolska Centre of Biotechnology, Jagiellonian University, Kraków, Poland; 3 Frankfurt Institute for Advanced Studies, Frankfurt am Main, Germany; 4 Institute of Biophysics, Goethe University Frankfurt, Frankfurt am Main, Germany; University of Oxford, UNITED KINGDOM

## Abstract

The steady emergence of SARS-CoV-2 variants gives us a real-time view of the interplay between viral evolution and the host immune defense. The spike protein of SARS-CoV-2 is the primary target of antibodies. Here, we show that steric accessibility to antibodies provides a strong predictor of mutation activity in the spike protein of SARS-CoV-2 variants, including Omicron. We introduce an antibody accessibility score (AAS) that accounts for the steric shielding effect of glycans at the surface of spike. We find that high values of the AAS correlate strongly with the sites of mutations in the spike proteins of newly emerging SARS-CoV-2 variants. We use the AAS to assess the escapability of variant spike proteins, i.e., their ability to escape antibody-based immune responses. The high calculated escapability of the Omicron variant BA.5 with respect to both wild-type (WT) vaccination and BA.1 infection is consistent with its rapid spread despite high rates of vaccination and prior infection with earlier variants. We calculated the AAS from structural and molecular dynamics simulation data that were available early in the pandemic, in the spring of 2020. The AAS thus allows us to prospectively assess the ability of variant spike proteins to escape antibody-based immune responses and to pinpoint regions of expected mutation activity in future variants.

## Author summary

Since the beginning of the COVID-19 pandemic, the steady emergence of new variants has thwarted the efficacy of vaccines and has made herd immunity a distant prospect. We show that steric accessibility to antibodies beyond the glycan shield is an excellent predictor of mutated positions in variants of concern, including Omicron. By using data from molecular dynamics simulations of a SARS-CoV-2 spike protein model constructed early in the pandemic, we demonstrate the power to predict sites of future mutation activity.

## Introduction

After an initial outbreak that affected the entire world, the COVID-19 pandemic caused by the severe acute respiratory syndrome coronavirus-2 (SARS-CoV-2) entered a new phase with the advent of the Omicron variants (BA.1 to BA.5). Of the several past and present variants of concern (VoC), Omicron BA.1 was the first escape variant, with significant reduction of immunity conferred by a previous infection with earlier variants or by vaccination [1, 2]. The induction of cross-neutralizing antibodies against Omicron infection from sera collected from patients previously infected with earlier VoC was strongly reduced [3]. Reduced vaccine effectiveness is alleviated by additional vaccination (“booster”) doses [4, 5]. Omicron BA.1 has 39 changes in the spike (S) protein counting each mutated, deleted, or inserted amino acid. Many of these changes are located in the receptor binding domain (RBD) mediating attachment to and entry into the cell through its interaction with the human angiotensin-converting enzyme 2 (ACE2) receptor.

Omicron has since further evolved. The main evolution route went through BA.2 into BA.4/BA.5. Here, we focus on the “original” Omicron (BA.1) and currently dominant strain in Europe, BA.5. Omicron BA.1 and BA.5 share 23 spike mutations. However, BA.5 lacks 14 mutations of BA.1 and has additional 11 mutations (https://covariants.org/variants/22B.Omicron; accessed 28 July 2022). As such, it shows a quite different mutation pattern with respect to BA.1. BA.5 likely descended from BA.2, harboring four additional mutations and lacking the Q493R mutation in BA.2.

An understanding of the factors driving the mutations in the spike protein and the ability to predict the sites of new mutations in future variants is of tremendous interest, given the impact of emerging variants on the trajectory of the pandemic [6]. Deep mutational scanning results indicate that the sublineages BA.2.12.1, BA.4 and BA.5 show neutralization evasion against both vaccination (with wild type spike) and from BA.1 infection [7]. This suggests that Omicron keeps evolving to evade the response to the previously prevalent variants [8].

A recent study used a deep mutational learning technique to assess the impact of RBD mutations on RBD-ACE2 binding from a yeast screening library [9]. The authors predicted a range of future antibody-escape RBD variants. Previously, Bai et al. predicted the effect of spike mutations from calculated binding free energies of complexes of spike with antibodies and the ACE2 receptor based on a coarse-grained model [10]. Chen et al. used short molecular dynamics (MD) simulations of spike variants to train a classification network in order to predict binding free energy changes of the RBD-ACE2 interactions [11]. Starr et al. used deep mutational scanning to systematically address RBD mutation effects on protein expression, ACE2 interaction, and antibody recognition [12, 13]. Recently, a structural investigation found that RBD-ACE2 binding affinity of the Omicron variant is similar to the Delta variant, and that Omicron spike displays significant antibody evasion [14], in line with earlier studies [1, 2, 15, 16]. Ovchinnikov & Karplus used a kinetic model of B-cell affinity maturation to determine the importance of bivalent versus monovalent antibody-antigen interactions in vaccination and infection [17].

Here, we first show that our earlier predictions of possible antibody-binding epitopes [18] align well with the sites of mutations in spike recorded in major spike variants. We then identify a simpler, yet more powerful predictor of mutation activity. The antibody accessibility score (AAS) uses MD trajectories of a fully glycosylated spike as input and reports the surface accessibility beyond the dynamic glycan shield. We show that the AAS captures sites of observed mutation activity in spike variants with high statistical significance, including the Omicron variants as well as earlier variants. The AAS provides us with a measure of the expected mutation propensity of all spike surface residues, including the RBD, in response to antibody-based immune pressure. As such, it should provide valuable information on future immune evasion.

## Results and discussion

### Spike mutations cluster in regions of predicted epitope candidates

In an earlier study [18], we used MD simulations of membrane-anchored SARS-CoV-2 spike [19] and bioinformatic tools to identify epitope candidates on spike. We defined a consensus epitope score [18] that integrated information on the surface accessibility of glycosylated spike, its structural rigidity, sequence variation at the onset of the pandemic, and a sequence-based bioinformatic epitope predictor. Applied to the MD simulation trajectories of spike, we identified nine distinct regions on the surface of spike with a high epitope score [18].

**Fig 1:**
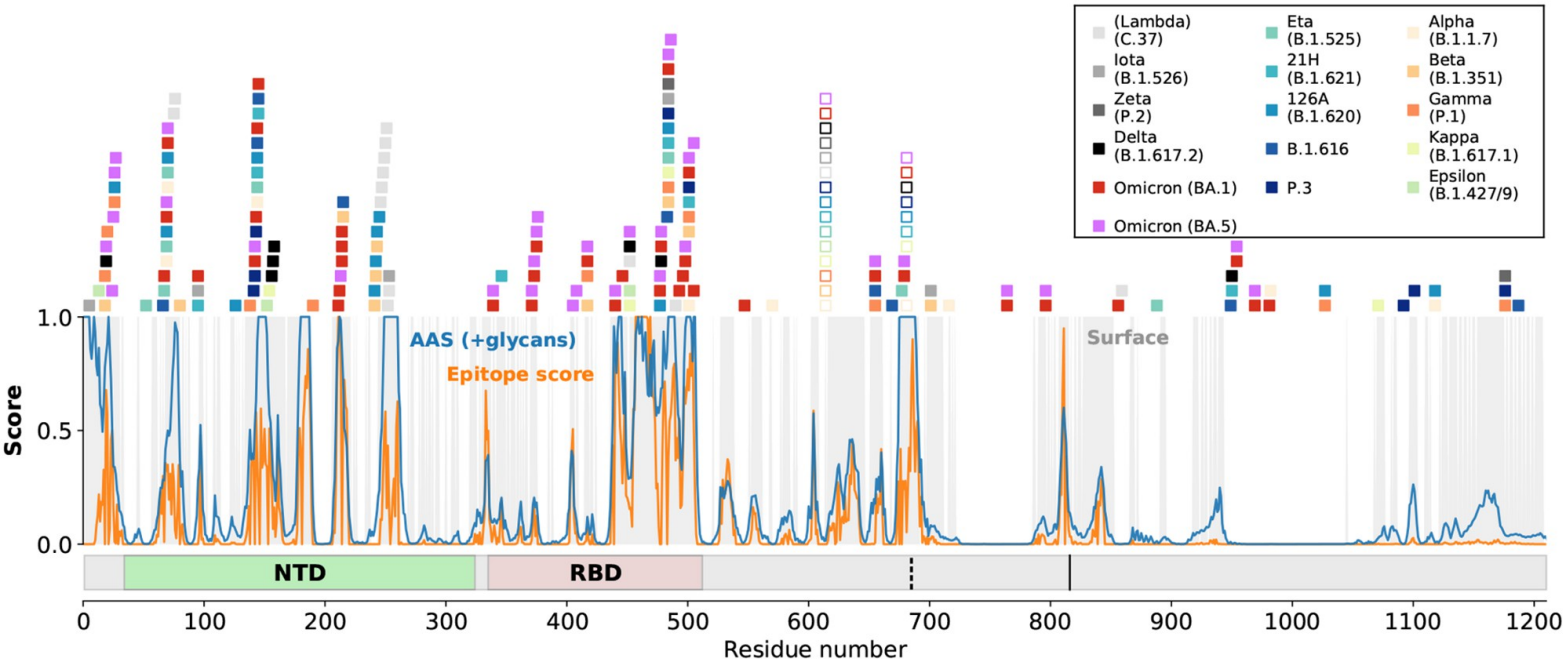
SARS-CoV-2 spike mutations and accessibility scores. Mutated residues in spike variants are marked by colored squares above the plot. Open squares indicate mutations at the furin cleavage site and D614G. The consensus epitope score of [18] is shown in orange, and the AAS considering glycans in blue. Grey shading in the plot indicates surface residues as listed in S1 Table. Variants are distinguished by color, as indicated in the legend. The N-terminal domain (NTD) and receptor-binding domain (RBD) are indicated at the bottom, as are the S1/S2 furin cleavage site (vertical dashed line) and the S2′ site (solid vertical line).

**Fig 2:**
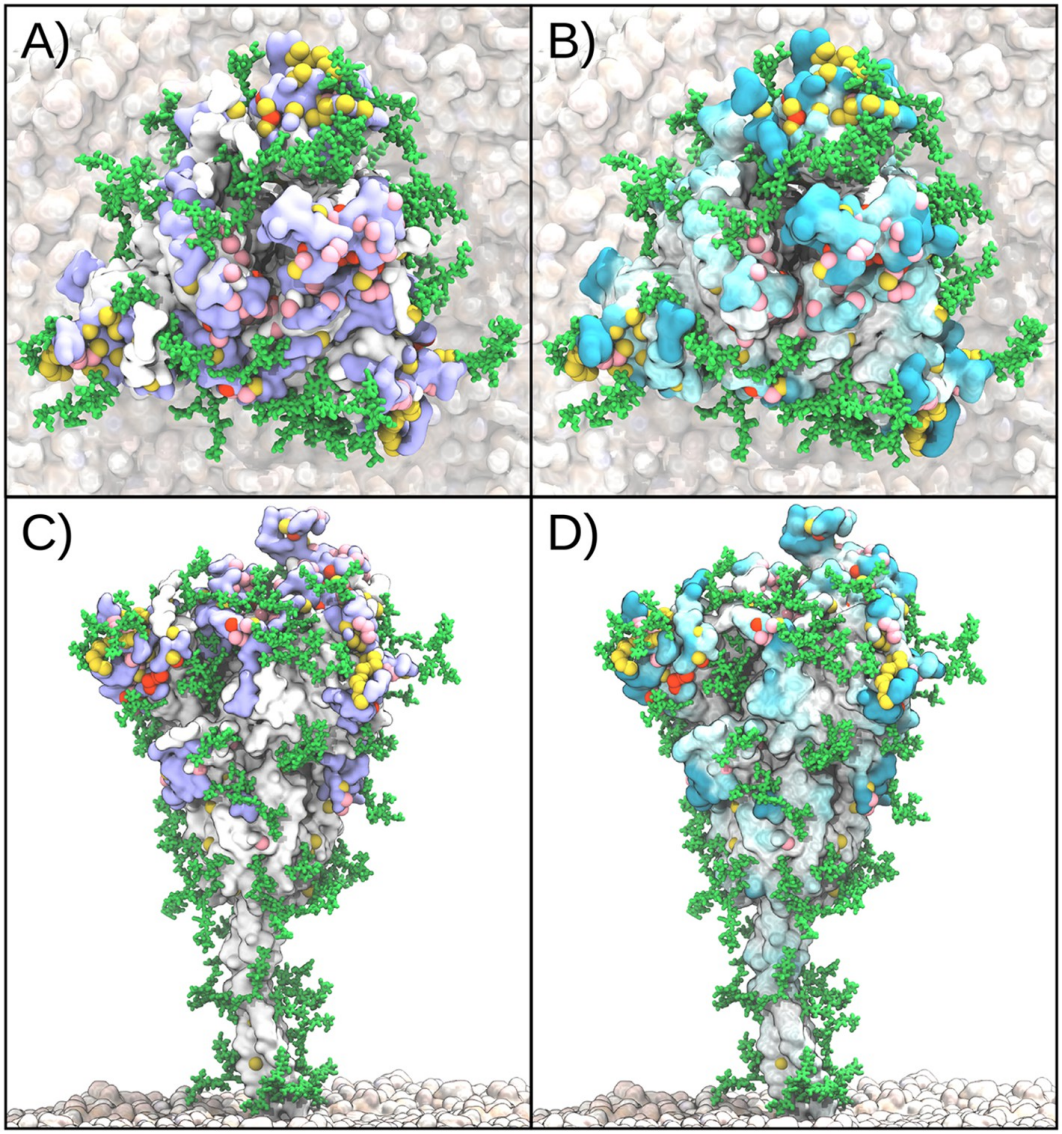
Mutations in the spike protein of SARS-CoV-2 variants concentrate in regions of high epitope and accessibility scores. Top views (A, B) and side views (C, D) of spike. Pink spheres show positions of Omicron BA.1 mutations, red spheres show positions unique to Omicron BA.5 mutations, and yellow spheres are those in earlier variants. Intense purple surface shading in A and C indicates high consensus epitope score [18]. Intense cyan surface shading in B and D indicates high AAS. Structures are snapshots from earlier MD simulations [19]. Proteins were rendered with Visual Molecular Dynamics (VMD) v1.9.3 [20].

The steady emergence of new SARS-CoV-2 variants allows us to test these computational predictions by comparing the reported sites of mutations in the spike protein of the variants with the predicted epitopes. Here, we assume that spike surface mutations are to a significant degree driven by antibody immune evasion. Throughout this work, we treat the Omicron variants and earlier variants separately, as defined in the Methods. By comparing the mutation sites in the SARS-CoV-2 variants to the regions with high predicted epitope score, we found that the first eight of the predicted nine epitope candidates are affected by mutations in Omicron BA.1 or at least one of the earlier variants (see Figs 1, 2A and 2C with renders created with Visual Molecular Dynamics (VMD) v1.9.3 [20]). Most of the mutations concentrate in the RBD and in the N-terminal domain (NTD); however, there is significant mutational activity also outside these regions.

**Fig 3:**
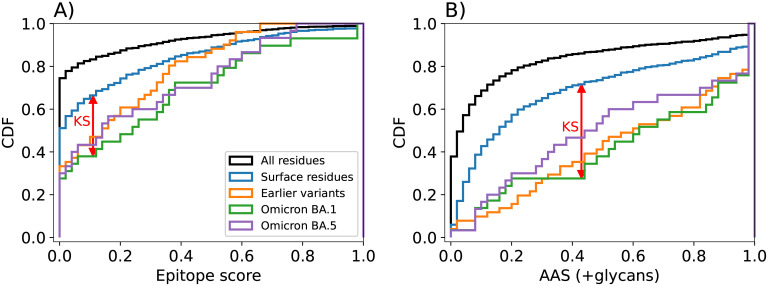
Epitope and antibody accessibility scores discriminate between spike mutation sites and generic surface residues. CDFs of (A) the epitope consensus score [18] and (B) the AAS considering glycans and calculated for all spike residues (black), surface residues (blue), sites of mutations in Omicron BA.1 (green), Omicron BA.5 (purple), and earlier variants (orange). Red arrows indicate the KS statistic as the maximum vertical gap between the CDFs for Omicron BA.1 mutation sites and surface residues, respectively. The corresponding P-values for the epitope score (A) and the AAS (B) are 0.016 and 1.5 × 10^−5^, respectively.

For a quantitative assessment of our epitope predictions in light of variant mutational activity, we compared the consensus epitope scores of mutated residues on the surface of spike to those of all surface residues. The cumulative distribution functions (CDFs) of the consensus epitope score reveal a significant shift towards higher scores for residues mutated in spike variants compared to all spike residues (Fig 3A); i.e., mutations occurred with high probability in regions identified as likely epitopes for antibody binding. The score distribution of mutation sites is also shifted significantly to larger values compared to surface residues on spike (for a definition of surface residues, see Methods, and S1 and S2 Tables). The P-values for the Kolmogorov-Smirnov (KS) statistic of Omicron BA.1 and earlier variants with respect to surface residues are 0.016 and 0.010, respectively, and thus significant. We conclude that a high epitope consensus score [18] is correlated with the occurrence of mutations in the spike protein of SARS-CoV-2 variants.

### Antibody accessibility score as predictor of emergent mutations in variants of concern

Next, we aimed to construct a simpler and more powerful predictor of mutation activity in variant spike based on surface accessibility alone, i.e., without including structural rigidity, sequence conservation, and sequence signatures. We compared three surface accessibility scores, “rays”, “antibody accessibility score” (AAS, termed “docking” in [18]), and the solvent-accessible surface area (SASA), with and without including surface glycosylation. The rays score quantifies the accessibility in terms of surface illumination by diffuse light [18]. The AAS measures how accessible each residue is to an antibody-fragment probe. SASA defines the solvent-accessible surface with a spherical probe (radius: 0.14 nm). All surface accessibility scores were determined for fully glycosylated spike structures collected during 4 × 2.5 μs MD simulations [19]. In this way, they account for the dynamic shielding by glycans [18]. In the following, we evaluate the different scores for variant mutation sites at the surface of spike and compare them to the scores of generic surface residues. Here, we assume that mutations in the interior of spike are driven by factors other than antibody binding.

The rays score without including surface glycosylation was used for the definition of general surface residues of spike. Compared to conventional surface definitions based on SASA, our rays analysis does not consider deep crevices as surface-exposed (S1 Fig). Thus, the estimated difference in score distributions of mutated residues versus surface residues is quite conservative in the sense that with a more generous definition, the inclusion of additional surface residues might result in even larger differences in the score distribution.

Consistently across variants, the AAS emerged as the top-performing score (Fig 3B, S2 Fig, and Table 1, see also Fig 2B and 2D for the distribution of AAS on the surface of spike). We used the KS statistic to quantify the power of the different scores to distinguish between sites of mutations on the surface of spike and general surface residues of spike. The P-values of the KS test for AAS with glycans with respect to all surface residues are substantially better than for the earlier epitope score. For Omicron BA.1 analyzed with AAS and glycans, we obtained a P-value of 1.5 × 10^−5^, and for the earlier variants a P-value of 2.3 × 10^−8^. By contrast, the simpler rays score was somewhat less discriminatory (S2A and S2B Fig, and Table 1). The commonly used SASA score was least suited to distinguish between mutated and general surface residues (S2E and S2F Fig), with an insignificant P-value of 0.31 for Omicron BA.1.

**Table 1:** Statistics of rays, antibody accessibility, and SASA scores in discriminating variant mutation sites from surface residues.

	Rays (−glycans)	Rays (+glycans)	AAS (−glycans)	AAS (+glycans)	SASA (−glycans)	SASA (+glycans)
KS(EV–S)	0.38	0.39	0.37	0.43	0.20	0.22
P(EV–S)	9.0 × 10^−7^	3.8 × 10^−7^	2.1 × 10^−6^	2.3 × 10^−8^	0.036	0.019
KS(BA.1–S)	0.36	0.43	0.28	0.45	0.18	0.19
P(BA.1–S)	8.0 × 10^−4^	3.1 × 10^−5^	0.021	1.5 × 10^−5^	0.31	0.25
KS(BA.5–S)	0.25	0.30	0.21	0.33	0.21	0.24
P(BA.5–S)	0.041	9.6 × 10^−3^	0.14	2.3 × 10^−3^	0.14	0.059
KS(BA.5-BA.1–S)	0.35	0.52	0.27	0.58	0.22	0.30
P(BA.5-BA.1–S)	0.015	4.2 × 10^−5^	0.10	1.8 × 10^−6^	0.27	0.064

Listed are the KS statistic and the corresponding P-value obtained for the distributions of the rays score, antibody accessibility score (AAS), and SASA score when comparing generic surface residues (S; see S1 Table) to surface mutation sites in Omicron BA.1 (S2 Table), Omicron BA.5, and earlier variants (EV). Scores were calculated with glycans (+ glycans) and without glycans (− glycans). Sample sizes *N* were 29 mutations for Omicron BA.1, 30 mutations for Omicron BA.5, 51 mutations for earlier variants, 19 surface mutations differing between BA.1 and BA.5, and 620 generic surface residues.

Omicron BA.5 shows slightly lower AAS value distributions (Fig 3B) compared to Omicron BA.1 and earlier variants. Remarkably, however, the set of residues differing between the BA.1 and BA.5 variants shows a strong shift towards high antibody accessibility for all metrics that include glycan shielding (Table 1, S2 Fig).

### The absence of glycan coverage is a determinant of mutation activity

Next, we investigated if glycosylation indeed plays a central role in steering the antibody-based immune defense, as we had assumed in the construction of the original epitope score [18]. In S2 Fig, we show the CDFs of the AAS and the rays score calculated without including glycosylation. In Table 1, we compare the KS statistic and the corresponding P-values as a measure of the discriminatory power of the AAS and rays score between sites of mutations at the surface of spike in major variants, and generic surface sites. We find that accounting for glycosylation substantially improves the discriminatory power of the AAS, and somewhat improves the discriminatory power of the rays score. For the mutation sites in Omicron BA.1, Omicron BA.5, and earlier variants assessed with the AAS against all surface sites, including glycosylation improved the P-value from 0.02 to 1.5 × 10^−5^, 0.14 to 2.3 × 10^−3^, and from 2.1 × 10^−6^ to 2.3 × 10^−8^. With the rays score and glycosylation included, the P-value of mutation sites improved from 8.0 × 10^−4^ to 3.1 × 10^−5^ in Omicron BA.1, from 0.041 to 9.6 × 10^−3^ in Omicron BA.5, and from 9.0 × 10^−7^ to 3.8 × 10^−7^ in earlier variants. Accounting for the dynamic glycan shield thus substantially improves the discriminatory power of the AAS, and to a lesser degree of the rays score.

### Impact of protein and glycan dynamics

To assess the impact of open and closed spike conformations on surface accessibility, the simulation was performed with chain A in the open conformation and chain B and C in the closed conformation [18, 19]. We calculated cumulative distributions of the rays score and AAS for different variants and methods for combining per-residue accessibility across the three protein chains: (i) maximum accessibility, (ii) sum of accessibility, (iii) product of accessibility. From (i) to (iii), the relative importance of less accessible chains increases. S3 Fig shows that pooling by the maximum accessibility results in the most robust differences in score distributions between variant and general surface residues. Therefore, method (i) is used throughout this work.

Next, we determined whether the sampling of protein and glycan dynamics is essential to discriminate variant mutation sites from generic surface residues. To this aim, we calculated the AAS and rays score for the static starting structure of the MD simulation with and without glycosylation. Here, we added glycans to the protein structure in a post-processing step with the software GlycoSHIELD [21]. GlycoSHIELD is used to attach glycan structures to proteins at predefined sequons by drawing glycan conformers from a simulation library [21]. Interestingly, we found that a single unglycosylated spike protein structure analyzed with the AAS or rays score suffices to distinguish between mutated residues and surface residues for Omicron BA.1 and earlier variants, but not for Omicron BA.5, with AAS P-values of 0.015 for Omicron BA.1, 0.29 for Omicron BA.5, and 1.6 × 10^−4^ for earlier variants (S4A and S5A Figs). Sampling of glycan motions improves all scores and allows us to distinguish between mutated residues and surface residues also for Omicron BA.5. We used GlycoSHIELD [21] (cutoff 3.5 Å, coarse-grained mode, glycan types as defined in [21]) to construct an ensemble of 160 spike models with different glycan conformers grafted onto the static protein structure. With this ensemble, we obtained AAS P-values of 9.0 × 10^−4^, 0.045, and 4.9 × 10^−7^, respectively, for Omicron BA.1, Omicron BA.5, and earlier variants (S4B and S5B Figs). As expected, we achieved the best performance by sampling both protein and glycan motions by taking the structures of the MD simulation (S4D and S5D Figs, and Table 1), with AAS P-values of 1.5 × 10^−5^, 2.3 × 10^−3^, and 2.3 × 10^−8^, respectively. Whereas expensive MD simulations are needed to get the best predictive power, mean field approaches such as GlycoSHIELD [21] still ensure accurate predictions at a fraction of the computational cost. This could prove valuable for high-throughput studies across a wide range of sequences and structural models for viral and non-viral targets.

### Immune escape mutations have high accessibility scores

**Fig 4:**
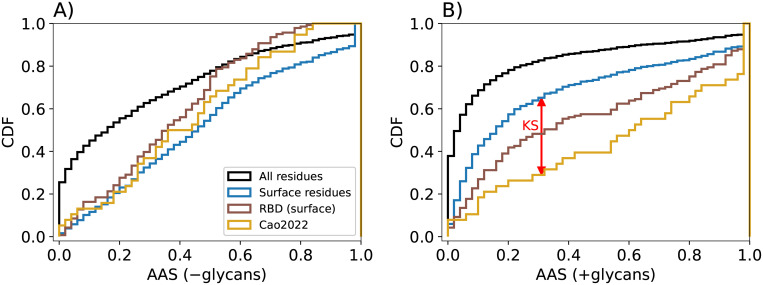
Immune escape mutations have high accessibility scores. CDFs of AAS are shown for mutations that were shown experimentally by Cao et al. [7] to reduce binding to neutralizing antibodies (gold). For reference, we show AAS distributions of surface residues (blue) and all residues (black) of spike, as well as all RBD surface residues (brown). Score distributions were calculated (A) without glycans and (B) with glycans. The KS statistic is the maximum vertical gap between the respective CDFs and is indicated by a red arrow in (B).

A recent experimental study [7] identified a set of spike mutations that severely weakened the binding of neutralizing antibodies. In Fig 4, we show that these mutation sites have a substantially higher AAS than general surface residues. With glycosylation considered, this shift results in a P-value of 4.6 × 10^−2^. Even if the analysis is restricted to the RBD, there is a noticeable shift of the mutation sites to higher AAS values compared to RBD surface residues (gold and brown lines, respectively, in Fig 4). We conclude that the comparably simple surface accessibility score presented here robustly identifies spike mutation sites important for immune escape.

Our findings here and earlier [18] complement a recent computational study that identifies the stalk domain as immunosubdominant due to the steric inability to recruit bivalently binding B-cell receptors [17]. Hindered accessibility of the conserved stalk covered by the glycan shield interferes with the development of broadly immunizing stalk antibodies. The kinetic model of antigen/B-cell binding affinities developed in ref. [17] could be combined with our surface accessibility quantification methods to rationalize why antibodies appear to target only relatively few SARS-CoV-2 epitopes after natural infection.

### Omicron differs from earlier variants in cumulative score

We also wondered whether Omicron differed from earlier variants in terms of the spike surface mutations. The Omicron variants have a large phylogenetic distance to other major variants and are not part of the antigenic cluster of previous VoC [3]. However, the distributions of the AAS of Omicron BA.1 and earlier variants in aggregate are similar (Fig 3B). In contrast, the AAS distributions of BA.5 are shifted to slightly lower values for most scores (S2 Fig). In a more fine-grained view (S2 Fig), we find similar accessibility score distributions for all variants. Thus, the accessibility of individual mutated residues in Omicron is, on average, not markedly different from those in earlier variants. What is different, however, is that the total number of mutations in the two Omicron variants considered here is larger, and thus the expected aggregate effect on immune evasion.

### Escapability of SARS-CoV-2 variants

We introduced an escapability score to quantify the aggregate effect of the mutations in a variant. The escapability score *F* aims to quantify the degree to which the mutations in a variant result in loss of binding to antibodies raised to a reference sequence of spike. As the predominant reference, we use WT spike, as in the first released vaccines. Motivated by our observation that the mutation sites concentrate in regions with high AAS, *F* measures the weighted fraction of antibody-accessible surface modified by mutations with respect to the reference sequence used, e.g., in vaccination. We extend the effect of a mutation to a 1-nm radius about the mutated site to account for the fact that mutations will perturb the structure and chemistry around the mutation sites. The escapability score *F* is defined in Eq 1 (Methods section) as the sum of the AAS *S*(*i*) over all residues *i* within 1 nm of a mutation site. For simplicity, we do not attempt to qualify the mutations by the change in amino-acid chemistry, which clearly are important for specific binding. For instance, a broad screen of antibodies revealed subtle dependences of their ability to neutralize the Omicron variants on mutations in the mapped epitopes [7]. By definition, the score of the WT spike is zero, and the highest possible escapability score is 1.0.

With this definition, we found that Omicron BA.1 and BA.5 have substantially higher escapability scores *F* compared to the other past and present VoC Alpha, Beta, Gamma, and Delta (Table 2 and S3 Table). Omicron variants differ from the earlier variants by having a substantially higher escapability score for chain A, which has the RBD in an open state in our model. Note that the RBD-opening of chain A breaks the symmetry between chains B and C, as reflected in slightly different escapability scores for the individual chains in Table 2. The increased escapability of Omicron BA.5 against WT antibodies is remarkable, given that the individual distribution is slightly shifted to lower scores (Fig 3B).

**Table 2:** Escapability score *F* of SARS-CoV-2 variants.

chain	Alpha	Beta	Gamma	Delta	BA.1	BA.5	BA.5 vs. BA.1
overall	0.18	0.26	0.25	0.23	0.52	0.48	0.40
A	0.18	0.22	0.22	0.19	0.52	0.48	0.40
B	0.18	0.26	0.25	0.23	0.41	0.46	0.31
C	0.17	0.18	0.16	0.17	0.43	0.39	0.33

The escapability scores calculated with AAS and glycans are listed for individual chains and overall.

The large value of *F* = 0.52 calculated with AAS and glycans for chain A of Omicron BA.1 compared to *F* ≈ 0.42 for chains B and C is due to the comparably large number of mutations localized in regions of the RBD that become exposed upon opening of the RBD for binding of the ACE2 receptor [14]. Similarly, the escapability of Omicron BA.5 in an Omicron BA.1 background is markedly different between chain A (open) and chains B and C (closed). By contrast, the escapability scores across the three chains are quite even in the Alpha, Beta, Gamma and Delta variants. The differences in inter-chain escapability scores in the Omicron variants point to the open state as a primary factor driving recent mutation activity. Given that the large number of mutations in Omicron spike barely improved its binding affinity to ACE2 [14], our premise that antibody-based immune defense drives the selection of spike mutations appears to hold also for mutations in the functionally important RBD.

We investigated if the recent Omicron variant BA.5 confers a predicted immune escape advantage over BA.1 following WT vaccination. Table 2 shows that BA.5 does not increase the calculated escapability following WT vaccination. We then tested if antibody escape from BA.1 infection in addition to WT vaccination could be a driving factor for the emerging variants BA.2 and BA.5. We therefore assessed the escapability of BA.5 with respect to the BA.1 variants and found a substantial escapability of BA.5 with respect to BA.1 as reference. Our data therefore indicate that—in addition to factors like infectiousness and incubation time—chemical difference in accessible residues with respect to the previously dominant variant supports antibody escape.

**Fig 5:**
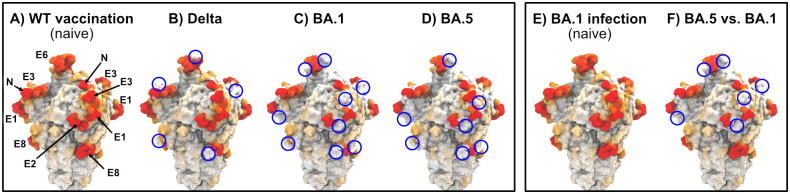
Mutations in emerging variants lower antibody access score on the surface of SARS-CoV-2 spike relative to WT and earlier variants. Spike renders with color intensity (white to red) indicating AAS, in which the AAS is set to zero in regions within 1 nm of a mutated site with respect to the reference sequence. (A, E): “Naive” antibody access by antibodies produced after (A) first WT vaccination (no previous infection) or (E) first infection by any variant (no previous vaccination). We assume here that all variants confer approximately the same accessibility to antibodies of naive hosts, and renders in (A) and (E) are identical. (B-D) Calculated reduction of AAS for antibodies produced against WT vaccination. (F) Calculated reduction of AAS for antibodies produced against BA.1 infection. Blue circles indicate notable reductions in AAS. Regions are labeled according to epitope candidates in [18]. N: N-terminus. In these renders, the AAS was set to zero for amino acids within 1 nm of any amino acid that was mutated with respect to the reference sequence.

To illustrate this point, we rendered the spike protein colored by the AAS. Fig 5 shows the reduction in AAS by variant mutations for antibodies produced against WT vaccination (Fig 5A) or against BA.1 infection (Fig 5E). In this visualization, the AAS was set to zero for all residues within a distance of 1 nm of any amino acid that differs from the reference sequence. Note that we assume the same surface accessibility to antibodies of naive hosts (without previous exposure to SARS-CoV-2 spike), irrespective of the spike variant. Panels Fig 5A and 5E are therefore identical. Spike variants BA.1 and BA.5 show more pronounced reductions in WT AAS than Delta (Fig 5B–5D). Further, BA.5 causes substantial calculated reductions in the AAS to BA.1 antibodies (Fig 5F).

## Conclusions

We showed that mutations in the spike protein of SARS-CoV-2 variants, including Omicron variants BA.1 and BA.5, concentrate in regions where access to antibodies is not hindered by glycans. A simple antibody accessibility score (AAS) distinguished sites of mutations in major spike variants from surface residues with a P-value <10^−7^ in a two-sample KS test. This contrasts to the P-value of 0.01 for our earlier more elaborate epitope score [18], which is thus significant but substantially less discriminatory even though it includes the AAS as one of its inputs. Already the simple rays score, which probes surface accessibility with diffuse illumination is more discriminatory with P-values <10^−6^. Based on the premise that immune evasion is a major driver of mutational activity in the SARS-CoV-2 spike, we conclude that MD-based computational epitope prediction can identify the regions targeted by neutralizing antibodies, with surface accessibility as its primary input. We also conclude that computational epitope prediction can provide guidance on sites of future mutation activity.

We introduced the escapability score *F* to quantify the aggregate effect of the mutations in a variant. *F* measures how much of the antibody-accessible surface has been modified by mutations when compared to the reference sequence used, e.g., in vaccination. For Omicron BA.1 compared to the WT spike, we find that with *F* ≈ 0.5 nearly 50% of the surface is modified, consistent with the observation of reduced protection against Omicron BA.1 by vaccination and previous infection with WT or a different variant [1, 2]. Omicron BA.5 shows similar escapability (*F* ≈ 0.5) as Omicron BA.1 from WT and strong escapability (*F* ≈ 0.4) from Omicron BA.1, suggesting substantial immune evasion of Omicron BA.5 against either WT vaccination/infection or Omicron BA.1 infection.

Our AAS and *F* scores strive for simplicity and leave out factors such as changes in chemistry as a result of the mutations. These could be quantified, for instance, by weighting the mutations with amino-acid substitution scores. Nevertheless, we find it encouraging that a fundamental physical feature—the steric access to an epitope unencumbered by glycans—emerges as a strong predictor of mutation activity in SARS-CoV-2 variants.

We also stress that the MD simulation model was based on limited structural and biological information available in the first few months of the COVID-19 pandemic [19]. Given the necessary shortcomings of the early MD simulation model and the fact that all major spike variants emerged after the initial simulation work, we consider the robust correlation between spike mutations and our surface accessibility scores to be promising.

In terms of the AAS, Omicron variants differ from earlier variants not by the type of mutations but by their number and overall impact. We found that the distributions of the AAS in Omicron BA.1 align well with those seen collectively in earlier variants, with Omicron BA.5 showing a slight shift to lower scores. However, in aggregate the Omicron mutations amount to a much larger escapability, as assessed by the escapability score *F*, against an immune response trained on spike of WT SARS-CoV-2 or earlier variants.

Our AAS and escapability scores provide the basis for estimating the impact of surface mutations on immune evasion from protein structural and dynamics data alone, complementary to sequence-based variant predictors. We have thus expanded the toolkit for anticipating possible future directions of mutational activity in the fight against the evolving virus.

## Methods

### Antibody accessibility score

The AAS is based on the “docking score” of [18]. As described in detail in [18], we performed extensive rigid-body docking of the Fab fragment of antibody CR3022 (PDB ID: 6W41 [22]) to probe the steric accessibility of the spike surface for antibody binding. In short, rigid-body docking of a coarse-grained model was performed using the simulation procedures described in [23, 24]. In the Monte Carlo docking simulations, we counted the contacts between residues of spike and the complementarity-determining region of the Fab. Structures that clashed with glycans were excluded in the calculation of scores “with glycans” (+ glycans). The counts were smoothed with a three-dimensional Gaussian filter (*σ* = 0.5 nm) based on the simulation starting structure. The resulting contact counts were then mapped linearly on the interval [0, 1] [18], yielding the AAS. The 5% and 95% percentiles were used for the lower and upper limit of the linear mapping.

### Rays accessibility score

The rays accessibility scores were based on quasi-isotropic illumination of the spike protein from light rays emanating from the surrounding of the protein, as described in detail in [18]. Glycans were considered (+ glycans) or not considered (− glycans) during illumination. The raw ray hits were used to define general surface residues (see below). For all other results in this paper, the rays scores were smoothed with a three-dimensional Gaussian filter (*σ* = 0.5 nm) and linear mapping on the interval [0, 1], as for the AAS.

### Spike surface residues

We defined the surface residues of spike based on the rays accessibility data of unglycosylated spike from [18]. We used a reference spike structure in which one RBD was open and two were closed in order to have both open and closed RBDs represented in a single ensemble. A cutoff of >1000 total ray hits across the 2.5 μs simulation was used for surface residues (S2 Fig). For reference, the maximum number of ray hits was about 5000. By imposing a minimum threshold of 1000 hits, we excluded crevices at the surface of spike from the surface definition (see S1 Fig). The definition of surface residues used here is thus conservative. We used the maximum value of ray hits across the three chains to define the surface accessibility of a particular residue, except in an analysis in which we distinguished between the chains B and C, in which the RBD was closed, and chain A, in which the RBD was open [18]. S1 Table lists the surface residues. We extended our analysis up to residue 1206 and excluded the transmembrane domain.

### Variant escapability function

The escapability *F*(*v*) of a SARS-CoV-2 variant *v* from modulation of dynamically accessible surface residues was defined as the sum of the antibody accessibility scores *S*(*i*) of all residues within 1 nm of a mutated residue,

$$\begin{array}{c} F\left(v\right)=\underset{c\in \{A,B,C\}}{\text{max}}[\sum_{i\in c}(S\left(i\right)[1-\prod_{j\in M(v)}\left(1-\theta_{ij}\right)])/\sum_{k\in c}S\left(k\right)] \end{array}$$ (1)

where the maximum is over the chains A, B, and C; the sums run over spike surface residues *i* and *k*, respectively; the product runs over the set *M*(*v*) of all mutated positions *j* in the three chains; and *θ*_*ij*_ = 1 if the two residues *i* and *j* are within a cutoff distance of 1 nm in the simulation starting structure, and *θ*_*ij*_ = 0 otherwise. The distance was measured at the residue centers-of-mass in the starting structure. The analysis is done separately for the three protein chains.

### Variants and mutation sites

In our analysis, we included Omicron and the earlier variants Alpha, Beta, Gamma, Delta, Epsilon, Zeta, Eta, Iota, Kappa, Lambda, Mu, B.1.620, B.1.616, and P.3. As the most recent VoC, we analyzed Omicron separately from the earlier variants. We used the main strain of Omicron (BA.1) and the recently emerged BA.5 sublineage in our analysis. Mutation sites present in multiple variants entered only once into the combined set of “earlier variants”. Deletions entered at the position of each deleted amino acid. Insertions entered with a single count at the position of the insertion. In the analysis, we included only the surface residues because mutations in the spike interior are unlikely to be driven by the evasion of the antibody immune response. We used the same surface definition as in the calculation of the AAS.

### Statistical assessment

We used the two-sample KS test to assess the significance of the differences in the distribution of epitope and accessibility scores. For this, we determined the KS statistic, *KS* = max_*S*_ |*P*(*s* < *S*) − *Q*(*s* < *S*)|, as the extremal distance of the respective CDFs of the scores, *P*(*s* < *S*) in surface sites and *Q*(*s* < *S*) in the respective set of variant mutation sites. Sample sizes are the number of surface residues and the number of mutation sites in the set, respectively. We report the KS statistic, the sample sizes, and the corresponding P-values in a two-sample KS test.

## Supporting information

S1 Table List of surface residues. Sites with mutations in at least one of the variants considered are listed in bold. Superscripts indicate the mutation count across the variants, as described in Methods.
(PDF)

S2 Table List of mutated residues on the surface of spike in the Omicron variants. (PDF)

S3 Table Escapability score *F* from different accessibility scores for past and present SARS-CoV-2 variants of concern. Escapability scores are calculated using Eq 1 with a cutoff of 1 nm and are reported for individual spike chains A, B, and C evaluated with the rays, AAS, and SASA scores with glycans (+ glycans) and without glycans (− glycans).
(PDF)

S1 Fig SARS-CoV-2 spike surface residues. Render of spike structure with surface residues colored in red. Surface residues are based on a cutoff of >1000 ray hits averaged over the atoms of each residue (see Methods in the main paper). The protein was rendered with VMD v1.9.3 [20].
(TIF)

S2 Fig Distribution of accessibility scores for surface residues of SARS-CoV-2 variants. Cumulative distribution functions (CDFs) of rays (A, B), AAS (C, D), and SASA (E, F) score calculated without (A, C, E) and with (B, D, F) considering glycans. CDFs of the scores are shown for all residues (black); surface residues (blue); and mutation sites at the surface of Omicron BA.1 (green), Omicron BA.5 (purple), and earlier variants (orange).
(EPS)

S3 Fig Distribution of accessibility scores from different combinations of individual chain scores. Cumulative distribution functions (CDFs) of rays (A, C, E) and AAS (B, D, F) with considering glycans. Per-residue scores of the three protein chains were combined by considering the maximum value across the chains (A, B), sum of values across the chain (C, D), or product of values across the chains (E, F). CDFs of the scores are shown for all residues (black); surface residues (blue); and mutation sites at the surface of Omicron BA.1 (green), Omicron BA.5 (purple), and earlier variants (orange).
(EPS)

S4 Fig AAS distributions for static and dynamic protein conformations. Cumulative distribution functions (CDFs) of static protein structure without glycosylation (A), with GlycoSHIELD glycosylation (B), and CDFs of fully dynamic simulated protein structure without glycosylation (C) and with glycosylation (D). CDFs of the scores are shown for all residues (black); surface residues (blue); mutation sites at the surface of Omicron BA.1 (green), Omicron BA.5 (purple), and earlier variants (orange). The KS statistic is the maximum vertical gap between the respective CDFs, as indicated by red arrows.
(EPS)

S5 Fig Rays accessibility score distributions for static and dynamic protein conformations. Cumulative distribution functions (CDFs) of static protein structure without glycosylation (A), with GlycoSHIELD glycosylation (B), and CDFs of fully dynamic simulated protein structure without glycosylation (C) and with glycosylation (D). CDFs of the scores are shown for all residues (black); surface residues (blue); mutation sites at the surface of Omicron BA.1 (green), Omicron BA.5 (purple), and earlier variants (orange). The KS statistic is the maximum vertical gap between the respective CDFs, as indicated by red arrows.
(EPS)
