# Supplementary material for: Antibody accessibility determines location of spike surface mutations in SARS-CoV-2 variants
Source: PLoS Comput Biol. 2023 Jan 24;19(1):e1010822. doi: 10.1371/journal.pcbi.1010822 (PMC9897577; doi:10.1371/journal.pcbi.1010822)
Supplement: S1 Table — Sites with mutations in at least one of the variants considered are listed in bold. Superscripts indicate the mutation count across the variants, as described in Methods. (PDF) [file pcbi.1010822.s001.pdf]

Table S1. List of surface residues.

**Spike surface residues**
1, 2, 3, 4, **5**^{1}, 6, 7, 8, 9, 10, 11, 12, **13**^{1}, 14, 15, 16, 17, **18**^{2}, **19**^{2}, **20**^{1},<br>21, 22, 23, **24**^{1}, **25**^{1}, **26**^{3}, **27**^{1}, 28, 29, 30, 32, 33, 45, 46, 57, 58, 59, 60, 61,<br>64, **66**^{1}, 68, **69**^{5}, **70**^{5}, 71, 72, 73, 74, **75**^{1}, **76**^{1}, 77, 78, 79, **80**^{1}, 82, 83,<br>85, 87, 88, 96, 97, 98, 99, 102, 108, 109, 111, 112, 113, 122, 123, 124,<br>125, 127, 129, 132, 134, 135, 136, 137, **138**^{1}, 139, 140, **141**^{1}, **142**^{3}, **143**^{2},<br>**144**^{6}, **145**^{3}, 146, 147, 148, 149, 150, 151, **152**^{1}, 153, **154**^{1}, 155, **156**^{1},<br>**157**^{1}, **158**^{1}, 159, 160, 161, 162, 163, 164, 165, 166, 167, 169, 170, 171,<br>172, 173, 174, 175, 176, 177, 178, 179, 180, 181, 182, 183, 184, 185,<br>186, 187, 188, 206, 207, 208, 209, 210, **211**^{1}, **212**^{1}, **213**^{1}, **214**^{1}, **215**^{2},<br>216, 217, 218, 219, 220, 221, 224, 226, 234, 236, 237, 244, **245**^{1}, **246**^{1},<br>**247**^{1}, **248**^{1}, **249**^{1}, **250**^{1}, **251**^{1}, **252**^{1}, **253**^{2}, 254, 255, 256, 257, 258, 259,<br>260, 261, 262, 269, 271, 272, 273, 278, 280, 281, 282, 284, 286, 287,<br>289, 292, 293, 294, 300, 305, 306, 307, 309, 310, 320, 321, 322, 323,<br>324, 325, 328, 330, 331, 332, 333, 334, 335, 336, 337, **339**^{2}, 340, 342,<br>343, 344, 345, **346**^{1}, 347, 348, 349, 351, 352, 354, 355, 356, 357, 359,<br>360, 362, 364, 366, 367, 370, **371**^{2}, 372, **373**^{2}, 374, **375**^{2}, 385, 388, 403,<br>404, **405**^{1}, 406, **408**^{1}, 409, 415, 416, **417**^{4}, 418, 420, 421, 424, 436, 437,<br>438, 439, **440**^{2}, 441, 442, 443, 444, 445, **446**^{1}, 447, 448, 449, 450, **452**^{5},<br>453, 454, 455, 456, 457, 458, 459, 460, 461, 462, 463, 464, 465, 466,<br>467, 468, 469, 470, 471, 472, 473, 474, 475, 476, **477**^{3}, **478**^{3}, 479, 480,<br>481, 482, **483**^{1}, **484**^{11}, 485, **486**^{1}, 487, 488, 489, **490**^{1}, 491, 492, **493**^{1},<br>494, 495, **496**^{1}, 497, **498**^{2}, 499, 500, **501**^{7}, 502, 503, 504, **505**^{2}, 506,<br>521, 527, 528, 529, 530, 531, 532, 533, 534, 535, 536, 537, 538, 551,<br>553, 554, 555, 556, 557, 558, 559, 560, 561, 568, 569, 577, 579, 580,<br>581, 582, 583, 584, 588, 590, 602, 603, 604, 605, 606, 607, 608, **614**^{15},<br>616, 617, 618, 619, 620, 621, 622, 623, 624, 625, 626, 627, 628, 629,<br>630, 631, 632, 633, 634, 635, 636, 637, 638, 639, 640, 641, 642, 643,<br>644, 645, 646, 651, 652, 653, 654, **655**^{4}, 656, 657, 658, 659, 660, 661,<br>668, 674, 675, 676, **677**^{1}, 678, **679**^{2}, 680, **681**^{8}, 682, 683, 684, 685, 686,<br>687, 688, 689, 690, 691, 696, 698, 699, 700, **701**^{2}, 702, 703, 704, 705,<br>706, 707, 708, 709, 710, 711, **716**^{1}, 717, 719, 721, 786, 787, 790, 791,<br>792, 793, 794, 795, **796**^{2}, 798, 799, 801, 803, 804, 807, 808, 809, 810,<br>811, 812, 813, 814, 815, 817, 820, 821, 823, 824, 825, 827, 828, 829,<br>830, 831, 832, 833, 834, 835, 836, 837, 838, 839, 840, 841, 842, 843,<br>844, 845, 846, 847, 848, 849, 850, 851, 852, 866, 867, 868, 869, 872,<br>891, 892, 893, 894, 895, 918, 919, 920, 921, 922, 925, 926, 928, 929,<br>932, 933, 935, 936, 937, 939, 940, 941, 942, 943, 1045, 1068, 1070, **1071**^{1},<br>1072, 1073, 1074, 1075, 1076, 1082, 1084, 1085, 1086, 1097, 1098, 1099,<br>1100, **1101**^{1}, 1102, 1103, 1110, 1111, 1112, 1114, 1122, 1124, 1125,<br>1126, 1127, 1128, 1130, 1131, 1132, 1133, 1134, 1135, 1136, 1138, 1139,<br>1140, 1142, 1143, 1144, 1146, 1147, 1149, 1150, 1151, 1153, 1154, 1155,<br>1156, 1157, 1158, 1159, 1160, 1161, 1162, 1163, 1164, 1165, 1166, 1167,<br>1168, 1169, 1170, 1171, 1172, 1173, 1174, 1175, 1177, 1178, 1180, 1181,<br>1182, 1184, 1185, **1187**^{1}, 1188, 1189, 1191, 1192, 1194, 1195, 1196,<br>1198, 1199, 1201, 1202, 1203, 1204, 1205, 1206, 1207

Sites with mutations in at least one of the variants considered are listed in bold. Superscripts indicate the mutation count across the variants, as described in Methods.
