# Supplementary material for: Antibody accessibility determines location of spike surface mutations in SARS-CoV-2 variants
Source: PLoS Comput Biol. 2023 Jan 24;19(1):e1010822. doi: 10.1371/journal.pcbi.1010822 (PMC9897577; doi:10.1371/journal.pcbi.1010822)
Supplement: S2 Table — (PDF) [file pcbi.1010822.s002.pdf]

Table S2. List of mutated residues on the surface of spike in the Omicron variants.

Variant	Omicron surface mutation sites
BA.1	69, 70, 142, 143, 144, 145, 211, 212, 214, 339, 371, 373, 375, 417, 440, 446, 477, 478, 484, 493, 496, 498, 501, 505, 614, 655, 679, 681, 796
BA.2	19, 24, 25, 26, 27, 142, 213, 339, 371, 373, 375, 376, 405, 408, 417, 440, 477, 478, 484, 493, 498, 501, 505, 614, 655, 679, 681, 764, 796, 954, 969
BA.4	3, 19, 24, 25, 26, 27, 69, 70, 142, 213, 339, 371, 373, 375, 376, 405, 408, 417, 440, 452, 477, 478, 484, 486, 498, 501, 505, 614, 655, 658, 679, 681, 764, 796, 954, 969
BA.5	19, 24, 25, 26, 27, 69, 70, 142, 213, 339, 371, 373, 375, 376, 405, 408, 417, 440, 452, 477, 478, 484, 486, 498, 501, 505, 614, 655, 679, 681, 764, 796, 954, 969
