# Supplementary material for: Antibody accessibility determines location of spike surface mutations in SARS-CoV-2 variants
Source: PLoS Comput Biol. 2023 Jan 24;19(1):e1010822. doi: 10.1371/journal.pcbi.1010822 (PMC9897577; doi:10.1371/journal.pcbi.1010822)
Supplement: S3 Table — Escapability scores are calculated using Eq 1 with a cutoff of 1 nm and are reported for individual spike chains A, B, and C evaluated with the rays, AAS, and SASA scores with glycans (+ glycans) and without glycans (− glycans). (PDF) [file pcbi.1010822.s003.pdf]

Table S3. Escapability score F from different accessibility scores for past and present SARS-CoV-2 variants of concern.

Method	Alpha	Beta	Gamma	Delta	BA.1	BA.5	BA.5 vs. BA.1
rays (−glycans)							
chain A	0.12	0.17	0.19	0.14	0.35	0.35	0.26
chain B	0.11	0.16	0.18	0.15	0.30	0.31	0.23
chain C	0.11	0.14	0.17	0.12	0.27	0.27	0.20
rays (+glycans)							
chain A	0.13	0.19	0.20	0.15	0.38	0.38	0.30
chain B	0.12	0.18	0.19	0.16	0.31	0.33	0.24
chain C	0.12	0.15	0.17	0.13	0.31	0.30	0.23
AAS (−glycans)							
chain A	0.14	0.14	0.16	0.13	0.35	0.34	0.25
chain B	0.13	0.18	0.19	0.17	0.31	0.33	0.23
chain C	0.12	0.13	0.15	0.11	0.27	0.26	0.20
AAS (+glycans)							
chain A	0.18	0.22	0.22	0.19	0.52	0.48	0.40
chain B	0.18	0.26	0.25	0.23	0.41	0.46	0.31
chain C	0.17	0.18	0.16	0.17	0.43	0.39	0.33

Escapability scores are calculated using eq. 1 with a cutoff of 1 nm and are reported for individual spike chains A, B, and C evaluated with the rays, AAS, and SASA scores with glycans (+glycans) and without glycans (−glycans).
